# COVID-19 Pandemic and Eating Disorders: What Can We Learn About Psychopathology and Treatment? A Systematic Review

**DOI:** 10.1007/s11920-021-01294-0

**Published:** 2021-10-21

**Authors:** Alessio Maria Monteleone, Giammarco Cascino, Eugenia Barone, Marco Carfagno, Palmiero Monteleone

**Affiliations:** 1 Department of Psychiatry, University of Campania “Luigi Vanvitelli,”, Naples, Italy; 2 Department of Medicine, Surgery and Dentistry ‘Scuola Medica Salernitana,’ Section of Neurosciences, University of Salerno, Salerno, Italy

**Keywords:** Eating disorders, COVID-19, Psychopathology, Risk, Treatment, Systematic review

## Abstract

****Purpose of Review**:**

This systematic review aims to collect evidence regarding the impact of the SarsCov-2 pandemic on people affected by eating disorders (EDs) targeting the following variables: psychopathology changes, mechanisms of vulnerability or resilience, and perception of treatment modifications during the pandemic.

****Recent Findings**:**

Since the beginning of the pandemic, a mental health deterioration has been detected in the general population and especially in people affected by pre-existing psychiatric conditions. Furthermore, mental healthcare has moved toward online treatment.

****Summary**:**

ED people showed a trend toward worsening of ED-specific psychopathology and impairment in general psychopathology. The most common vulnerability mechanisms were social isolation and feelings of uncertainty, while heightened self-care and reduced social pressure were resilience factors. The online treatment, although raising many concerns related to its quality, was considered the best alternative to the face-to-face approach. These findings may support the idea that stressful events contribute to the exacerbation of ED psychopathology and highlight the relevance of internalizing symptoms in EDs. The identification of putative risk and resilience variables as well as of subjective factors affecting online treatment perception may inform healthcare professionals and may promote more personalized approaches.

## Introduction

The coronavirus disease-2019 (COVID-19) pandemic has worldwide affected human physical and mental health [1, 2]. Several studies have detected negative effects of the pandemic on mental health in the general population [3], and the WHO declared that addressing mental health during the pandemic is a priority [4••, 5]. People affected by pre-existing psychiatric conditions were even more vulnerable to the COVID-19 infection and to develop psychiatric sequelae [6••, 7]. Previous studies from past similar outbreaks revealed that psychiatric sequelae persisted after the acute event in people at risk [8].

The COVID-19 pandemic is a traumatic event, which encompasses several types of stressors, including fear of contagion, worries for relatives’ health, social distancing and isolation, disruption in routine activities and in everyday life, and change in the economic status [9–11]. It could be conceived as a huge psycho-social stressor with multifaceted components, and Vinkers et al. [12] suggested the opportunity for researchers to examine strategies to successfully deal with stress and adapt to the new circumstances.

People affected by eating disorders (EDs) have been considered at high risk during the COVID-19 pandemic [13•]. Indeed, since the beginning of this event, researchers have raised many concerns regarding the possible negative effects of the pandemic on ED individuals [14], since people with EDs are highly sensitive to social stress [15] and uncertainty [16] and have high need of control and difficulties in regulating emotions [17]. Rodgers et al. [18•] hypothesized that these individuals would have been vulnerable to the COVID-19 pandemic because of their sensitivity to disruption in daily activities and restrictions, the heightened exposure to ED-specific media messages, and their difficulty to manage fear of contagion. In the light of previous data related to people who had been quarantined in the SARS outbreak occurring in 2003 [19], an increase not only in ED-specific symptoms but also in post-traumatic stress symptoms may be hypothesized in this population. In addition to the putative psychopathology exacerbation, the researchers have also hypothesized several changes in the routine diagnostic and care strategies, including the management of medical problems resulting from their abnormal eating behaviors, discontinuation of day-hospital programs, and limitations in the access to face-to-face or group treatments with the consequent urgent need to adapt at and transit to online delivered treatments [13•, 14, 20–22]. Further concerns have been added regarding the accessibility of e-health services and the quality of therapeutic alliance through telemedicine [13•]. It is also worth considering that the COVID-19 pandemic has posed an increased burden on healthcare professionals [23•, 24], who need evidence-based recommendations in addition to those adapted from the pre-pandemic evidence [13•]. However, no study to date has collected literature evidence regarding the impact of the COVID-19 pandemic on psychopathology and treatment of people with EDs.

This systematic review aims to gather evidence from studies regarding the impact of the COVID-19 pandemic on people affected by EDs exploring (1) changes in ED-specific and general psychopathology; (2) mechanisms of vulnerability and resilience to the COVID-19 pandemic exposure; and (3) change in treatment delivery service, in terms of the patients’ perception of online treatment, potential barriers and/or advantages of this method, and its effectiveness.

## Methods

### Information Sources and Searches

The PRISMA guidelines were followed to select and assess published articles [25].

In order to perform a systematic review of the literature, the following search keys were used in PubMed: “(COVID) AND (((eating) AND (disord*)) OR (anorexia) OR (bulimi*) OR (bing*))”. Bibliographies from relevant papers were inspected to identify studies not yielded by the initial search.

### Eligibility Criteria

Articles were selected according to the following inclusion criteria: the paper (1) was a peer-reviewed research article published in English; (2) included samples of people with a current or lifetime diagnosis of any ED; and (3) was published between January 1st, 2020, and April 30th, 2021. Review papers, meta-analyses, commentary, study protocols, and case reports were excluded.

### Study Selection and Data Collection Process

**Fig. 1:**
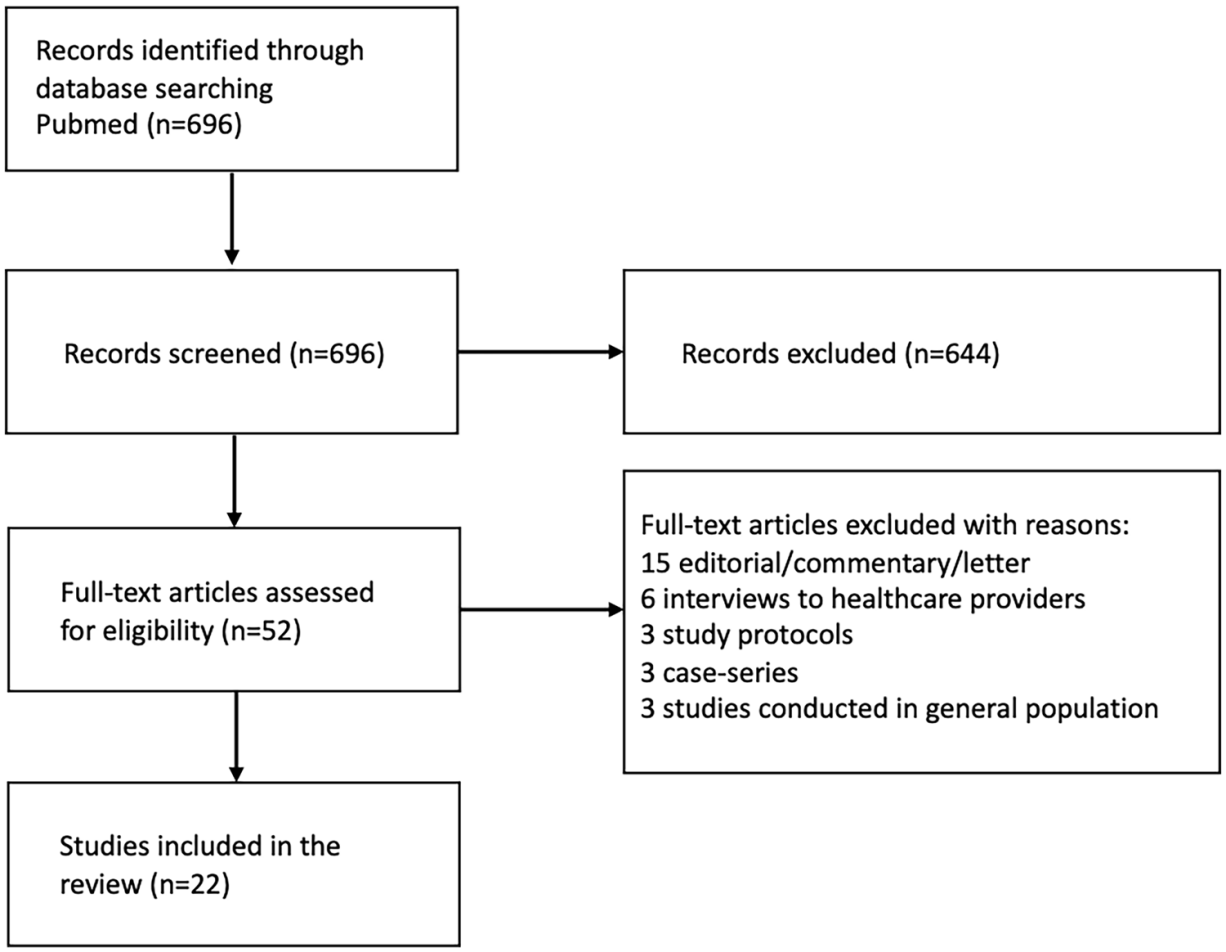
Flow diagram of the study inclusion

The literature search identified 696 papers, which were screened against the inclusion criteria. Fifty-two full-texts were assessed. Thirty studies were excluded because they did not meet the eligibility criteria: fifteen were editorial/commentary/letter, six were interview of healthcare providers or caregivers, three were study protocols, three were case series, and three were conducted on general population. This resulted in the inclusion of 22 studies in the qualitative synthesis. Figure 1 reports the flow diagram of study inclusion.

## Results

### General Characteristics of Selected Studies

All the studies were conducted during the first wave of SarsCov-2 pandemic.

Most studies (14 of 22) were quantitative studies, 4 showed a quantitative–qualitative design, and 4 were qualitative studies. The main characteristics (diagnosis, sample size of each patients’ group, and diagnosis), the assessed outcomes, and the main findings of quantitative studies are reported in Table 1. The main qualitative findings studies are reported in Table 2.

**Table 1:** Description of included studies with quantitative methodology

Study	Sample	Diagnosis	Age (years) mean (SD)	Study design	Outcome and measures	Findings
Baenas et al. [43]	74 adults, 95.9% female	19 AN 12 BN 10 BED 33 OSFED	32.12 (12.84)	Cross-sectional, no comparison with a control group	Factors associated with psychopathology worsening (EDI-2, YFAS-2, SCL-90-r, TCI-R, self-developed survey)	25.7% reported symptoms worsening Low self-directedness was associated with a deterioration in ED symptoms and general psychopathology
Branley-Bell and Talbot [41]	129, 93.8% female	Self-reported ED (80.6% currently ill)	29.27 (8.99)	Cross-sectional, no comparison with a control group	Psychopathology (SWEMWBS, PSS, ESSI, SCI, RRS-ED)	86.7% reported that their symptoms had worsened 30% reported that their symptoms were much worse The recovery group showed higher mental wellbeing, lower perceived stress, higher social support, and higher perceived control
Castellini et al. [30]	74 adults with ED	37 AN 37 BN 97 HC	31.7 (12.8) 30.5 (10.9)	Longitudinal, comparison with a control group	Psychopathology (BSI, EDE-Q, IES-R) Factors associated with ED psychopathology worsening during COVID-19 lockdown (CTQ, ECR-R, self-developed survey) Factors associated with general psychopathology worsening during COVID-19 lockdown (CTQ, ECR-R, self-developed survey)	ED patients vs HC: greater worsening of binge eating, compensatory behaviors, and post-traumatic stress symptoms ED patients, pre- vs post-COVID-19: worsening of physical exercise Household arguments (with physical exercise) Fear for safety of the loved ones (with binge eating) Childhood trauma and insecure attachment style with post-traumatic stress symptoms
Fernández-Aranda et al. [14]	32 EDs, 90.6% female	13 AN 10 BN 5 OSFED 4 BED	29.2 (range 16–49)	Cross-sectional, no comparison with a control group	Psychopathology (self-developed survey)	38% (12 out of 32) reported impairments in their ED symptomatology 56.2% (18 out of 32) reported additional anxiety symptoms
Fernández-Aranda et al. [39]	87 EDs, 89,7% female	55 AN 18 BN 14 OSFED	33.7 (15.8)	Cross-sectional, no comparison with a control group	Psychopathology (CIES) Treatment change	People with AN and BN showed an improvement in eating symptoms and emotion regulation, while people with OSFED worsened in anxiety and depressive symptoms post-confinement People with AN showed less satisfaction of online treatment
Graell et al. [31]	365 patients, 87.9% female	255 AN 48 ARFID 26 BN 37 OSFED	Outpatients:14.74 (2.33) Day-hospital: 13.18 (3.03)	Mixed: cross-sectional and longitudinal, no comparison with a control group	Psychopathology (not specified)	41.9% of patients reported reactivation of eating symptoms; adolescents presented a more pronounced reactivation of ED and non‐ED symptoms than children 68.2% of patients and their families identified the onset of confinement as a possible precipitating factor for admission 31.8% of adolescents reported increase in family conflicts 40.9% of patients reported social isolation from peers Compared to those admitted in 2019, the hospitalized patients in 2020 were affected by more frequent comorbidity, affective disorders, and suicide risk
Leenaerts et al. [28]	15 females	BN	Median (Q1–Q3): 23 (21.5–25.5)	Longitudinal, experience sampling method, no comparison with a control group	Psychopathology (PANAS, self-assessment of eating episodes)	No change in binge-purging behaviors frequency before–during lockdown Increase of negative affect and decrease of positive affects before–during lockdown. A higher binge eating frequency during the lockdown was linked to stronger changes in negative affect
Lewis et al. [45]	63 ED patients, 91% female	24 AN 20 BN 16 BED 3 other EDs	27 (11.47)	Cross-sectional	Treatment change (self-developed survey) Factors associated with positive perceptions toward online treatment (self-developed survey)	40% agreed that the transition to online treatment adversely affected the effectiveness/quality of their treatment 68% stated that they would not prefer continuing online therapy given the choice 54% stated that they would not recommend online treatment Longer duration in treatment, strength of therapeutic alliance, fear of COVID-19
Machado et al. [32]	43 ED patients, 95.3% female	20 AN 14 BN 2 BED 7 OSFED	27.6 (8.45)	Longitudinal, no comparison with a control group	Psychopathology (EDE-Q, CIA, UPPS-P, DERS, CIS)	No changes (eating symptoms, impulsivity, psycho-social impairment) pre-during confinement Most participants considered that COVID-19 changed moderately to extremely their life in terms of routines, stress experienced, physical exercise and eating habits
Monteleone et al. [33]	320 ED patients, 93.8% female	179 AN 63 BN 48 BED 22 OSFED	29.19 (12.05)	Retrospective, no comparison with a control group	Factors associated with ED psychopathology worsening during COVID-19 lockdown (self-developed survey with items selected and adapted from EDI-2, GAD-7, PHQ-9, PTSD Checklist for DSM-5, OCI) Factors associated with general psychopathology worsening during COVID-19 lockdown (self-developed survey with items selected and adapted from EDI-2, GAD-7, PHQ-9, PTSD Checklist for DSM-5, OCI)	Positive association: heightened isolation and fear of contagion Negative association: perceived therapeutic relationship quality, satisfaction with family relationships and friends’ relationships Positive association: heightened isolation and fear of contagion Negative association: perceived therapeutic relationship quality, satisfaction with family relationships and friends’ relationship, entrusting of others
Monteleone et al. [34]	320 ED patients, 93.8% female	179 AN 63 BN 48 BED 22 OSFED	29.19 (12.05)	Retrospective, no comparison with a control group	Psychopathology: before vs lockdown periods (self-developed survey with items selected and adapted from EDI-2, GAD-7, PHQ-9, PTSD Checklist for DSM-5, OCI) Psychopathology: lockdown vs post-lockdown periods (self-developed survey with items selected and adapted from EDI-2, GAD-7, PHQ-9, PTSD Checklist for DSM-5, OCI)	Anxiety, depression, stress, post-traumatic stress symptoms, obsessive–compulsive symptoms, insomnia, panic symptoms, suicide ideation, ineffectiveness, impulsivity, social insecurity, body dissatisfaction, and self-induced vomiting were significantly higher during lockdown Anxiety was significantly higher in the post-lockdown Suicide ideation, social insecurity, binge eating, physical activity, and body dissatisfaction were significantly lower in the post-lockdown
Nisticò et al. [35]	59 ED patients, 96.6% female	22 AN 15 BN 22 BED 43 HC	30.1 (12.9) 34.7 (12.7)	Longitudinal, comparison with a control group	Psychopathology (DASS-21, IES-R, PSS, 5 questions from the EDE-Q)	ED patients vs HC: higher eating symptoms, stress, anxiety, depression, avoidance, intrusion, hyperarousal, and perceived stress score Lockdown vs post-COVID-19: patients scoring significantly lower during post-COVID19 in intrusion, hyperarousal, losing control over food, thinking about body, seeing body. No change in stress, anxiety and depression scores
Phillipou et al. [36]	180 adults, 95.6% female	Self-reported past or current ED: 88 AN 23 BN 6 BED 4 OSFED 68 not specified	30.5 (8.2)	Cross-sectional, no comparison with a control group	Psychopathology (survey adapted from the EDE-Q and the DASS-21)	In the ED group: 64.5% reported a little or a lot more food restriction, 35.5% reported increased binge eating behaviors, 18.9% reported increased purging behaviors, 47.3% reported increased exercising In the AN subgroup: 67.1% reported increased restricting behaviors, 20.5% reported increased binge eating, 18.2% reported increased purging, 48.9% reported increased exercise
Schlegl et al. [26]	55	BN	24.42 (6.36)	Cross-sectional, no comparison with a control group	ED Psychopathology (self-developed survey) General psychopathology (self-developed survey) Treatment change Coping strategies (self-developed survey)	49.1% reported a worsening of their ED symptomatology, 40.0% reported new symptoms, 47.3% reported increased binge eating, 36.4% reported increased self‐inducing vomiting, 9.1% reported increased laxative use 7.3% of patients reported increased diuretic abuse Sadness, loss of energy, inner restlessness, and loneliness were the most pronounced depressive and general psychopathology symptoms (over 75%) 81.8% of patients received face-to-face therapy before the COVID‐19 pandemic compared to 36.4% during the pandemic Most used: enjoyable activities, virtual social contact with friends and mild physical exercises
Schlegl et al. [27]	159	AN	22.42 (8.67)	Cross-sectional, no comparison with a control group	ED psychopathology (self-developed survey) General psychopathology (self-developed survey) Treatment change Coping strategies (self-developed survey)	41.5% agreed that their symptoms had gotten worse, 20% reported new symptoms, 70% reported increased ED cognitions, more than 60% reported increased physical activity Comparison between adults and adolescents: adults were slightly more affected and reported a greater impairment of therapy than adolescents More than 70% reported that loneliness, inner restlessness, and sadness increased 50% indicated fears of not being able to stop or control worries and worries that feelings get out of control. 46.6% reported increases in family conflicts Patients receiving in-person outpatient psychotherapy decreased from 88.1 to 55.3%, weighing by a clinician or a therapist from 48.4 to 30.8%, and visits at the general practitioner from 44.0 to 23.9% 10.7% of patients that had been receiving therapy before the pandemic did not get any therapy during the pandemic Most used: daily routines, day planning, enjoyable activities, and mild physical activities
Shaw et al. [46]	12 patients	Not specified	Not collected	Cross-sectional	Treatment change (self-developed survey adapted from ESQ)	Increase in the number of admissions of ED patients to the general pediatric ward Face-to-face was the preferred type of appointment No change in service satisfaction between before and during the COVID-19 pandemic
Termorshuizen et al. [37•]	1021, 98% female	Self-reported ED history: 665 AN 203 Atypical AN 295 BN 216 BED 192 OSFED 108 Other	US sample: 30.61 (9.37)	Cross-sectional, no comparison with a control group	Psychopathology (self-developed survey to assess ED symptoms and the GAD-7) Factors associated with psychopathology worsening (self-developed survey)	79% (US, N = 397) and 66% (NL, N = 331) of respondents were concerned about worsening of the eating disorder due to lack of structure. Fur- thermore, respondents were concerned about worsening of the eating disorder due to being in a triggering environment (US 58%; NL 57%) or lack of social support (US 59%; NL 48%), and being unable to access food consistent with their meal plan (US 61%; NL 36%) 57% reported feeling anxious about not being able to exercise, over one third of participants reported worsening of dietary restriction and compensatory behaviors 71.3% were concerned about worsening of the eating disorder due to a lack of structure, 57.5% were concerned about worsening of the eating disorder due to being in a triggering environment, 53.5% were concerned about lack of social support, and 48.6% about being unable to access food consistent with their meal plan 79% (US, N = 397) and 66% (NL, N = 331) of respondents were concerned about worsening of the eating disorder due to lack of structure. Fur- thermore, respondents were concerned about worsening of the eating disorder due to being in a triggering environment (US 58%; NL 57%) or lack of social support (US 59%; NL 48%), and being unable to access food consistent with their meal plan (US 61%; NL 36%) 57% r79% (US, N = 397) and 66% (NL, N = 331) of respondents were concerned about worsening of the eating disorder due to lack of structure. Fur- thermore, respondents were concerned about worsening of the eating disorder due to being in a triggering environment (US 58%; NL 57%) or lack of social support (US 59%; NL 48%), and being unable to access food consistent with their meal plan (US 61%; NL 36%)
Vuillier et al. [38]	207, 63.3% female	Self-reported ED: 91 AN 46 BN 44 BED 26 OSFED	30.0 (9.7)	Cross-sectional, no comparison with a control group	Psychopathology (EDE-Q, DASS-21) Factors associated with psychopathology worsening (DERS)	83.1% reported worsening of ED symptomatology, without differences between diagnostic subgroups Emotion regulation difficulties, such as having fewer strategies, poorer emotional clarity, and non-acceptance of emotions, explained nearly half of the variance in emotional distress during the pandemic

*AN* anorexia nervosa, *BED* binge eating disorder, *BN* bulimia nervosa, *BSI* brief symptom inventory, *CTQ* childhood trauma questionnaire, *CIA* clinical impairment assessment, *CIES* COVID isolation eating scale, *CIS* coronavirus impact scale, *DASS* depression, anxiety and stress scale, *DERS* difficulties in emotion regulation scale, *ECR-R* experience in close relationships-revised, *EDE-Q* eating disorders examination questionnaire, *EDI-2* eating disorders inventory-2, *EDs* eating disorders, *ESSI* ENRIHD social support instrument, *ESQ* experience of service questionnaire, *GAD-7* generalized anxiety disorder 7-item scale, *IES-R* impact of event scale-revised, *OCI* obsessive–compulsive inventory *OSFED* other specified feeding or eating disorder, *PANAS* positive and negative affect schedule, *PHQ-9* patient health questionnaire 9, *PSS* perceived stress scale, *PTSD* post-traumatic stress disorder, *RRS-ED* rumination response scale for eating disorders, *SCI* Shapiro control inventory, *SCL-90-R* symptom checklist-90-revised, *SWEMWBS* short Warwick-Edinburgh mental Wellbeing scale, *TCI-R* temperament and character inventory-revised, *UPPS-P* impulsive behavior scale-negative urgency subscale, *YFAS-2* Yale food addiction scale-2

**Table 2:** Description of included studies with qualitative methodology

Study	Sample	Outcome	Findings
Branley-Bell and Talbot [41]	129 patients with self-reported ED	Factors associated with psychopathology worsening Factors associated with psychopathology improvement Treatment change	Changes in normal living situation due to the pandemic have worsened ED symptoms Most of the sample reported greater feelings of social isolation as a result of the pandemic. A lack of routine and/or distractions created more time for rumination about weight, exercise habits, and meals Participants reported spending more time online with increased exposure to triggering messages Using the Internet and social media to speak to friends, support from ED communities, reduced social comparisons Participants reported being prematurely discharged from in-patient units, having treatment suspended or remaining on a waiting list for treatment While online support was described as a positive factor, participants described this as falling short of treatment and support received in-person
Brown et al. [44]	15 patients with self-reported ED	Factors associated with psychopathology change Treatment change	Social isolation was associated with increased eating disorder behaviors Increase in accountability was associated with improvements in eating disorder behaviors Increased responsibility was associated with both improvement and worsening of eating disorder behaviors Lack of routine and need for intentionality were associated with increased eating disorder behaviors Participants compared personal health concerns with overall health concerns surrounding COVID-19 pandemic: they believed their situation was not as critical, but nevertheless required more attention than was offered Participants had different experiences regarding online services
Clark Bryan et al. [42]	21 patients with AN	Psychopathology Factors associated with psychopathology change Treatment change	Participants reported heightened anxiety related to both the lockdown and the exercise, and increased obsessive–compulsive behaviors. They described ED behaviors as a source of control and reassurance Disruption in routine and lack of activities providing control and distraction, associated with an increased uncertainty Participants reported a reduced access to eating disorder services and increased attempts at self-management in recovery
Frayn et al. [29]	11 patients with BED	Psychopathology Factors associated with psychopathology worsening Treatment change	Participants reported both symptom deterioration and improvement Factors surrounding social distancing and stay-at-home measures were found to both improve and worsen symptoms for different patients Patients reported positive perceptions of tele-therapy, describing this modality as facilitating attendance and engagement
McCombie et al. [40]	32 patients with a current or recovered self-reported ED	Factors associated with psychopathology worsening Factors associated with psychopathology improvement	Isolation, low mood, anxiety, rumination, disruption to routines, and media/social media messages around weight and exercise Having more space and time for healing and self-care, perceiving less pressure to engage in social activities, improved relationships
Shaw et al. [46]	43 participants: 12 patients 19 parents/carers 12 staff members	Treatment change	Patients, parents/carers, and staff all preferred face-to-face appointments over virtual options. Patients experiences technological barriers and difficulties to “open up”; they felt the video sessions “less real” and reported less pressure from the services
Termorshuizen et al. [37•]	1021 with self-reported ED	Psychopathology Factors associated with psychopathology improvement	Participants reported increased suicidality and substance use, fear to gain weight, and to not exercise enough Participants reported positive effects including increase in social support, greater connection with family, more time for self-care, and motivation to recover
Vuillier et al. [38•]	207 with self-reported ED	Factors associated with psychopathology worsening Factors associated with psychopathology improvement Treatment change	Participants reported experiencing a greater level of distressing emotions (fear and/or uncertainty) with a negative impact on their ED. Changes to routine during the pandemic resulted in more accessibility to food and exercise, as well as increased time and/or flexibility to engage in ED behaviors. Participants who were living alone described feeling confined and isolated. Participants reported exposure to unhelpful social messages (transformation and diet) Lack of work and social pressure, creating boundaries to look after self, adding in positive activities (e.g., oil painting, photography, different forms of writing) Patients described their experience of support as being of a lesser quality thanks to their usual support for the following reasons: not having a confidential space at home, the quality of the internet connection, a less personal connection with the therapist In contrast, some patients commented on their experience of having a strong therapeutic relationship and described the treatment as more accessible and the lack of support as an opportunity to take more responsibility

*AN* anorexia nervosa, *BED* binge eating disorder, *ED* eating disorder

All the selected studies, except those by Schlegl et al. [26•, 27•], Leenaerts et al. [28], and Frayn et al. [29], were conducted in mixed ED samples. Fifteen studies were conducted in patients with a current ED, 4 were in mixed samples with a current or a past ED, and 3 studies were conducted in samples with a self-reported diagnosis of an ED (1 current diagnosis, 2 current or past diagnosis). Five studies conducted a longitudinal assessment, although four of these [28, 30••, 31, 32] compared levels of symptomatology during the pandemic with those in the pre-pandemic period. The remaining studies adopted a cross-sectional design (e.g., asking participants if their symptoms had changed during the pandemic period), with the exception of 2 studies [33•, 34•] which conducted a retrospective evaluation of psychopathology. Only two studies [30••, 35] compared symptomatology levels between patients and healthy controls. Twelve studies reported that patients with EDs were in treatment, 5 did not report this data, and 3 studies were conducted in both treated and untreated patients and 2 in recently discharged patients. Three studies included outpatients, and 3 study included both inpatients and outpatients.

Seven studies reported the prevalence of SarsCov-2 infection among patients with EDs ranging from 0 to 5%.

### COVID-19 Related Eating Disorder Psychopathology Effects

Most studies [14, 26•, 27•, 30••, 31, 33•, 36, 37•, 38•] identified a significant impairment in ED core symptoms (i.e., food restriction, binge-purging behaviors, and physical exercise). Considering the studies adopting a descriptive procedure, we identified a worsening of ED symptomatology occurring in a range from 38 [14] to 83% [38•] of the assessed samples. However, no change in the severity of symptomatology was found in other two studies adopting a longitudinal design [28, 32], while an improvement in eating symptoms was observed by Fernandez-Aranda et al. [39]. Worsening in the severity of symptomatology did not differ between patients with a current ED diagnosis and those with a lifetime diagnosis in two studies [37•, 40] but not in Branley-Bell and Talbot [41] who reported greater impairment in those currently ill. The studies [26•, 27•] conducted in people with a single diagnosis (AN or BN) found that almost 50% of the recruited samples reported ED symptom worsening. These studies [26•, 27•] also highlighted that when ED-related cognitions were evaluated, the impairment was even more common than that of behavioral ED symptoms, occurring in 70%, 80%, and 87% of samples with AN [27•], with BN [26•], or with mixed ED diagnoses [41], respectively. A general worsening of ED-related cognitions was also found in other studies [33•, 35]. Across ED-related aberrant behaviors, physical exercise is worth of a specific mention. Indeed, the possibility to do physical activity was reduced as result of pandemic restrictions: this promoted a widespread increase of anxiety related to inactivity effects [30••, 33•, 37•, 42] with high variability [41] in the amount of physical exercise performed by the patients.

When differences between the main ED diagnoses were investigated, a greater concern about food restriction was found in AN individuals, while more frequent binge eating was detected in the BN ones, suggesting that differences between the ED diagnoses are consistent with diagnostic characteristics [37•]. Differences between people with AN and those with BN were identified also by Castellini et al. [30••] who found that the latter group was more vulnerable to the pandemic restrictions because of their interference with the recovery process. On the other hand, three different research groups [33•, 36, 38•] failed to identify an effect of the diagnosis on the ED symptom trajectory during and after the pandemic lockdown, although the comparisons were conducted between AN individuals and mixed ED groups.

Only two studies [30••, 35] compared ED symptom impairment between people with EDs and healthy controls by employing a longitudinal approach. Castellini et al. [30••] found that the intensity of symptom (i.e., objective binge eating and physical exercise) worsening was significantly greater in patients than in controls. Nisticò et al. [35] found that the severity of ED symptom decreased in the re-opening period following the first lockdown (March to May 2020). This was consistent with the results of another study [33•] adopting a retrospective design and highlighting that in the re-opening period, the ED symptoms returned to the levels seen before the lockdown.

Limitations of these studies need to be acknowledged. First, except for Schlegl et al.’s study [27•], differences between adults and adolescents were not assessed: this precludes the possibility to predict age-related vulnerability to EDs during the COVID-19 pandemic. Second, only two studies adopted a prospective design and included a comparison group, and a few studies included patients with a clinically defined diagnosis. Third, most of the studies did not assess differences across the main ED diagnoses: this limits the possibility to draw transdiagnostic conclusions.

### COVID-19-Related General Psychopathology and Quality of Life Effects

Changes in general psychopathology during the lockdown were assessed in 11 studies. Three of them focused on specific psychopathology variables and revealed an increase in anxiety [14, 37•] and post-traumatic stress symptoms [30••] during the lockdown period. A more comprehensive evaluation of several internalizing symptoms was conducted in the remaining studies [26•, 27•, 28, 29, 33•, 35, 42]. Overall, these studies agreed that people with EDs experienced heightened anxious and depressive symptoms during the lockdown. Schlegl et al. [26•, 27•] identified loneliness, sadness, and inner restlessness as the most pronounced general symptoms in AN and BN people with 70–75% of the assessed patients reporting a deterioration of these symptoms. Remarkably, a longitudinal design was employed in three of these studies [28, 30••, 35]. Furthermore, Monteleone et al. [33•] and Nisticò et al. [35] found that the worsening of internalizing symptoms persisted in the re-opening period that followed the first lockdown in Italy. Furthermore, an increased rate of comorbidity, affective disorders, and suicide risk was observed in children and adolescents recovered for their ED in the first months of the 2020 in comparison to those hospitalized in the same period of the previous year [31]. However, it is worth to outline that only two studies [30••, 35] adopted a prospective design and a comparison with a control group, while only Monteleone et al. [33•] included a large sample of people with EDs.

The quality of life perception was evaluated in three studies through a quantitative assessment [26•, 27•, 32]. Reduced satisfaction was observed in 62% of BN individuals and in 50% of AN people discharged from previous hospital admission [26•, 27•], while no significant change was reported by Machado et al. [32] who evaluated the ED-induced clinical impairment.

### Predictors and Correlates of COVID-19-Related Psychopathology Changes

Predictors of symptom change during the COVID-19 lockdown period were evaluated in three studies adopting a quantitative design [30••, 34•, 43]. Two of these studies [30••, 43] pointed to low self-directedness, childhood traumatic experiences, and insecure attachment as predictors of the COVID-19-related ED symptoms deterioration [43] and post-traumatic stress symptoms onset [30]. In a large population with mixed ED diagnoses, the path analysis [34•] showed that heightened isolation and fear of contagion predicted ED and general symptom worsening as well as reduced satisfaction with family and with friends’ relationships and reduced perceived social support were associated with ED and general symptoms deterioration, respectively. The quality of the therapeutic relationship was a resilient factor for people with EDs [34•].

The factors related to the COVID-19 psychopathology worsening were assessed in 8 qualitative studies [29, 31, 37•, 38•, 40–42, 44]. Social restrictions, negative emotions, changes in routine, and thin-related social media messages were described as possible factors contributing to mental health deterioration in most of those studies. Heightened social isolation was reported in all the qualitative studies. Negative emotions included heightened rumination and anxiety [29, 38•, 40]; changes in routine activities encompassed disruption in living situation, which promoted hiding their ED from others and increased pressure from relatives to eat more [40, 41, 44], more free time with boredom and lack of distraction [38•, 40, 41], reduced opportunities to exercise [38•, 41], change in food availability at home [37•, 38•, 41], and increased intentionality and responsibility in planning their own actions [44]. These studies pointed to perceived uncertainty and lack of control as the common mechanisms by which the disruption in routine activities promoted psychopathology deterioration in ED people during the COVID-19 lockdown. However, routine changes [29, 44] and social isolation [29] were sometimes associated with symptom improvement. In this line, useful strategies helping patients to face with COVID-19-related distress were detected and can be divided in two groups: heightened self-care and reduced pressure to engage in social activities or reduced social/work pressure [29, 38•, 40, 41, 44]. The former included increased focus and responsibility for recovery [37•, 44], creating boundaries to look after self [38•], time spent in enjoyable activities/hobbies, or mild physical exercise [26•, 27•, 38•, 42].

The main limitation of the qualitative studies is their small sample sizes. Furthermore, a few studies have evaluated the effects of personality-related characteristics and of theoretically suggested variables (i.e., early abuse) that may contribute to explain the observed variation in psychopathology trajectories.

### COVID-19-Related Treatment Effects

The main COVID-19-induced treatment change was a reduced access to in-person treatment [26•, 27•, 41, 42, 45]. Schlegl et al. [26•] found that the rate of BN patients receiving face-to-face treatment decreased from 82 to 36% during the lockdown. The parallel increase of online treatment was often perceived as characterized by impairment in the quality of the therapy [26•, 27•, 37•, 38•, 41, 42, 44–46]. In this line, Lewis et al. [45] also reported that 54% of the ED sample would not recommend the online treatment and 68% would not choose to continue the online therapy. Positive predictors of a good perception of the online therapy were longer illness duration, higher COVID-19-related anxiety, and stronger therapeutic relationship [45]. Fernández-Aranda et al. [39] found that the patients with AN were those reporting lower satisfaction with the online transition. On the other hand, a positive perception of the online therapy was reported in some other studies [29, 44, 46], and there is evidence that patients who interrupted all kinds of treatment were those showing the highest symptom worsening during the lockdown [37•]. In this line, the online treatment allowed patients to maintain a strong and safe therapeutic relationship [38•] and made treatment more accessible for some patients [29, 38•, 46]. In another study, no effect of the treatment delivery strategy (i.e., direct access or telehealth) was found on the psychopathology worsening experienced during the lockdown [34•]. The main barriers identified by the patients regarding the online treatment were perceiving a detached connection with the therapist [38•, 39, 46]; technological difficulties (e.g., low quality of Internet connection or lack of private space) [38•, 46]; and concerning about self-monitoring due to reduction of the therapist’s pressure that patients need to resist the demands of the illness [41, 46]. Overall, the online treatment was described as the best alternative when face-to-face therapy was not available [38•, 41, 46]. Finally, a few studies found that individuals with EDs described their need for mental care as less important than that for physical care related to the COVID-19 infection and perceived themselves as an unjustified burden on the health system [38•, 41, 44, 46]. It is worth mentioning that the comparison between face-to-face and telehealth therapies as well as the treatment successful rates during the pandemic has been not sufficiently explored. Although previously recommended [13•], no evaluation of self-help treatment effectiveness has been provided.

## Discussion

This systematic review assessed the impact of the COVID-19 pandemic on people with EDs. A trend toward worsening of ED-specific psychopathology with respect to the pre-pandemic period was observed as well as an impairment in general psychopathology. Feeling of uncertainty was the putative common mechanism promoting mental health deterioration in individuals with EDs, although resilience mechanisms such as supporting interpersonal relationships and heightened self-care emerged. The treatment has largely moved toward online delivering strategies that, despite being considered by patients as the best alternative to the face-to-face approach, were affected by concerns about the quality of the online therapy. A wide variation in both psychopathology changes and perception of the quality of treatments has been observed among individuals with EDs.

Regarding the effects of the COVID-19 pandemic on psychopathology, it is worth noting that ED-specific symptoms deterioration was often observed, although data were even more consistent when referring to the general psychopathology (e.g., anxiety or depressive symptoms) worsening. No differences across the main ED diagnoses were identified, although they were not deeply investigated. These data are corroborated by the increase in urgent and routine referrals of individuals with EDs and their relatives [47] as well as by the increase of in-patient admissions for EDs especially observed in adolescents [48–50]. This evidence may support the hypothesized post-traumatic nature of ED symptomatology, as previously suggested in experimental [51, 52] and review studies [53]. Indeed, the data collected during the pandemic have been replicated across different samples exposed to the same stressful condition, providing novel and reliable evidence of a transdiagnostic vulnerability to acute stress. More severe internalizing symptoms, primarily anxiety and depressive symptoms, were also found during the pandemic in people with EDs. Interestingly, there is some evidence [33•, 35] that their worsening persisted even in the re-opening period which followed the first lockdown, while ED-specific symptoms returned to the pre-pandemic levels. Heightened anxiety during this period may reflect the sensitivity to societal pressures which characterizes people with EDs [54]. These findings are also consistent with the widespread reported onset and/or exacerbation of affective symptoms observed during the pandemic in people with pre-existing psychiatric conditions [23•, 55]. However, they also support theoretical models [16, 56–58] and literature [59] describing affective symptoms as core symptoms of ED psychopathology.

It is worth noting that studies reported that some individuals with EDs remained stable in their symptoms during the lockdown, while others even improved. The inconsistency of these findings may be the result of the heterogeneity of the study methodologies: most of them included mixed ED samples with patients at different illness phases (i.e., currently ill, recovered, or discharged from hospitalization) or different treatment conditions (i.e., face-to-face or online) and different diagnostic evaluation processes (i.e., self-reported assessment of the ED diagnosis or clinically defined diagnosis). However, these findings also highlight the variability of the patients’ response to such an acute challenge and provide interesting data regarding mechanisms of resilience or illness deterioration. Although causal interpretation may be limited by the correlation nature of most of the study results, the high number of qualitative studies included in this review contributes to overcome this issue. The lack of interpersonal relationships providing security feelings and support as well as negative emotions and uncertainty feelings was the most common mechanisms making individuals with EDs more vulnerable to the COVID-19 pandemic. They were promoted from the disruption in routine activities (e.g., reduced time spent with friends and more with household members, familiar conflicts, increased exposure to diet-related social media messages) associated with the COVID-19-related restrictions. Unlike other psychiatric conditions [23•] and initial expectations [13•, 60], no effect of the economic condition was found on the mental health of people with EDs. On the other hand, developing new routines and planning positive (e.g., distracting) activities and having more space and time to healing and self-care and less pressure to engage in social activities were useful strategies to face with the pandemic restrictions. These findings corroborate the hypothesis that ED-related behaviors can be conceived as maladaptive coping strategies to face with emotional distress [61–63] and may inform clinicians about the therapeutic need to develop adaptive emotional coping strategies to promote recovery from EDs. In line with Brown et al. [44], it is also possible to suggest that the effects of restrictions may change in the light of patients’ living and work situations. This highlights the importance to consider the subjective context surrounding patients’ illness.

The effects of the COVID-19 pandemic were observed also on the treatment. In addition to the well-known transition to the online treatment that involved all psychiatric disorders [64], this systematic review highlights that the face-to-face treatment still represents the preferred modality for individuals with EDs and that the online therapy is considered the best alternative. These findings support previous suggestions in EDs [65, 66•]. Concerns related to the telemedicine approach were related to the perception of the therapeutic relationship as more detached and impersonal as well as to some technologic barriers. However, as for the psychopathological trajectory during the pandemic, also the perception of online treatment changed across individuals with EDs, who also described this treatment as promoting more accessibility to therapies, as an opportunity to heightened and more responsible self-management of the illness and to maintain a good therapeutic relationship. COVID-19-related findings confirm the role of the therapeutic alliance as one of the most important resilience factors for individuals with EDs [67]. Finally, treatment-related data revealed a sort of self-stigma given that many patients reported feelings of guilt or being undeserving of treatment in comparison to the need of physical healthcare due to the COVID-19 disease. This is in line with the internalized stigma seen in individuals with EDs [68] and with stigma-related data for other psychiatric conditions collected during the pandemic [69] and may contribute to worsen the renowned unmet treatment needs among people with EDs [70].

## Conclusions

The COVID-19 pandemic induced several psycho-social stressors in people with EDs. Despite exacerbation of ED-specific symptomatology and deterioration of general psychopathology have been observed during this period, great variability exists among people affected by these illnesses. In this line, the identification of factors promoting variability in psychopathological change as well as in the perception of online treatment may inform researchers and healthcare professionals. Clinicians are advised to target interpersonal and emotion regulation difficulties of people with EDs and their subjective response to stressful events as well as to consider the patient’s experience of online treatments and to identify his/her potential barriers to this approach. These findings may meet the suggested need [71–73] for a more targeted and individualized approach for people with EDs. Finally, they can contribute to develop protocols promoting early diagnosis, recommendations for patients and therapists, and instruments to manage such an emergency period and the phase that follows.
